# Health-related quality of life after mandibular resection for oral cancer: Reconstruction with free fibula flap

**DOI:** 10.4317/medoral.19399

**Published:** 2014-03-08

**Authors:** Wenli Yang, Sanjun Zhao, Fei Liu, Minglei Sun

**Affiliations:** 1 Department of Stomatology, The First Affiliated Hospital of Zhengzhou University, Zhengzhou,China; 2 Deparrtment of Prosthodontics, School of Stomatology, Fourth Military Medical University. Xi´an, China

## Abstract

Objectives: Mandibular resection for oral cancer is often necessary to achieve an adequate margin of tumor clearance. Mandibular resection has been associated with a poor health-related quality of life (HRQOL), particularly before free fibula flap to reconstruct the defect. The aim of this study was to evaluate health-related quality of life in patients who have had mandibular resections of oral cancer and reconstruction with free fibula flap. Study Designs: There were 115 consecutive patients between 2008 and 2011 who were treated by primary surgery for oral squamous cell carcinoma, 34 patients had a mandibular resection. HRQOL was assessed by means of the 14-item Oral Health Impact Profile (OHIP-14) and University of Washington Quality of Life (UW-QOL) questionnaires after 12 months postoperatively. Results: In the UW-QOL the best-scoring domain was mood, whereas the lowest scores were for chewing and saliva. In the OHIP-14 the lowest-scoring domain was social disability, followed by handicap, and psychological disability. Conclusions: Mandible reconstruction with free fibula flap would have significantly influenced on patients’quality of life and oral functions. The socio-cultural data show a fairly low level of education for the majority of patients.

** Key words:**Health-related quality of life, free fibula flap, mandibulectomy, UW-QOL, OHIP-14.

## Introduction

Free fibula flap as a source of vascularized bone has gained widespread use since its first description by Taylor in 1975 (1). Compared to other free flaps, the free fibula flap offers the greatest bone length, a single vascular pedicle of sufficient length with large diameter vessels and rich periosteal blood supply; this allows multiple osteotomies to bridge large mandibular defects across the midline, the option for a skin paddle of intermediate thickness and the opportunity to operate simultaneously at the donor and recipient sites (2).

It is generally agreed that patients with mandibular invasion by oral squamous cell carcinoma should be treated surgically. A mandibular resection is required in patients with significant mandibular invasion. Immediate reconstruction is preferable, and with the growing familiarity with free fibula flap, particularly for the anterior defect (3). However,mandibular resection has long been associated with a poor quality of life.

Successful reconstruction has often focused on the rate of survival of free flaps rather than on patients’ quality of life. Health-related quality of life (HRQOL) has become an increasingly important outcome measure for patient’s undergoing treatment for a wide array of illnesses. It is by definition multi-dimensional and reflective of the patient’s point of view (4).

Little information exists in the literature regarding the patients’ HRQOL after mandibular resections. Hence, the purpose of our study was to evaluate by questionnaire the HRQOL of patients who have had mandibular resections of oral cancer and reconstructions with free fibula flap.

## Patients

Because this study was retrospective it was granted an exemption in writing by in the First Affiliated Hospital of Zhengzhou University of Ethical Review Board. The study cohort was composed of 115 consecutive patients between 2008 and 2011, who were treated by primary surgery for oral squamous cell carcinoma (SCC), 34 patients had a mandibular resection. All patients with significant mandibular invasion and need immediate reconstruction with free fibula flap. For the purposes of this investigation, patients with tumors arising from the upper jaw were excluded as were patients with oropharyngeal SCC, non-SCC malignancies, and patients who previously had had any treatment of any modality. Other inclusion criteria were: free flap survived completely; age less than 65 years; no previous or synchronous malignancies; no cognitive impairment; at least 12 months after reconstruction; patients with recurrence of the disease were not excluded.

- Questionnaires and data collection

The most recently the University of Washington Quality of Life (UW-QOL) questionnaire was used in this study. The UW-QOL scale is filled in by the patient and provides a broad measure of QOL for patients with head and neck cancer with good acceptability, practicality, validity, reliability, and responsiveness (5,6).The questionnaire is composed of 15 domains: 12 are disease-specific items (pain, appearance, activity, recreation, swallowing, chewing, speech, shoulder, taste, saliva, mood, and anxiety), and 3 are global questions. Each of the 12 included questions has 3-6 response options. The domains are scored on a scale ranging from 0 (worst) to 100 (best).

Besides the 15 questions, patients were asked to choose no more than 3 of the 12 disease-specific domains that had been the most important to them in the preceding 7 days. We scored the individual domains according to the UW-QOL guidelines. The standard UW-QOL is available as a Chinese version and has been validated for a Chinese population (7).

OHIP-14 consists of 14 items divided into 7 different domains: functional limitation, physical pain, psychological discomfort, physical disability, psychological disability, social disability, and handicap. Each item is scored as: 0 = never; 1 = hardly ever; 2 = sometimes; 3 = fairly often; and 4 = very often. The domains are scored on a scale ranging from 0 (best) to 100 (worst). The higher the score, the poorer the patient’s state of health. The standard OHIP-14 is available as a Chinese version and has been validated for a Chinese population (7).

- Statistical analysis

Data were recorded, and then analyzed with the help of the Statistical Package for the Social Sciences (SPSS version16.0, SPSS Inc., IBM). Probabilities of less than 0.05 were accepted as significant.

## Results

Thirty-four patients with oral cancer were included in this analysis, all patients completed the questionnaire when back to the hospital regularly review compliance. Of the 34 patients who completed questionnaires, there were 25 men and 9 women with a median age of 53.4 (range 28–65); The alveolus(N=13, 38.23%) and floor of mouth (N=9, 26.47%) were the most common sites ( Table 1). Followed by buccal mucosa (N=7,20.59%) and tongue (N=5,14.71%). In terms of location of mandibular resection, 32.35% were located in the anterior, 23.53% in posterior, and 44.12% combined. Ten patients of 33(30.30%) were classified as T1–T2, while 23 (69.70%) were classified as T3–T4.

**Table 1:**
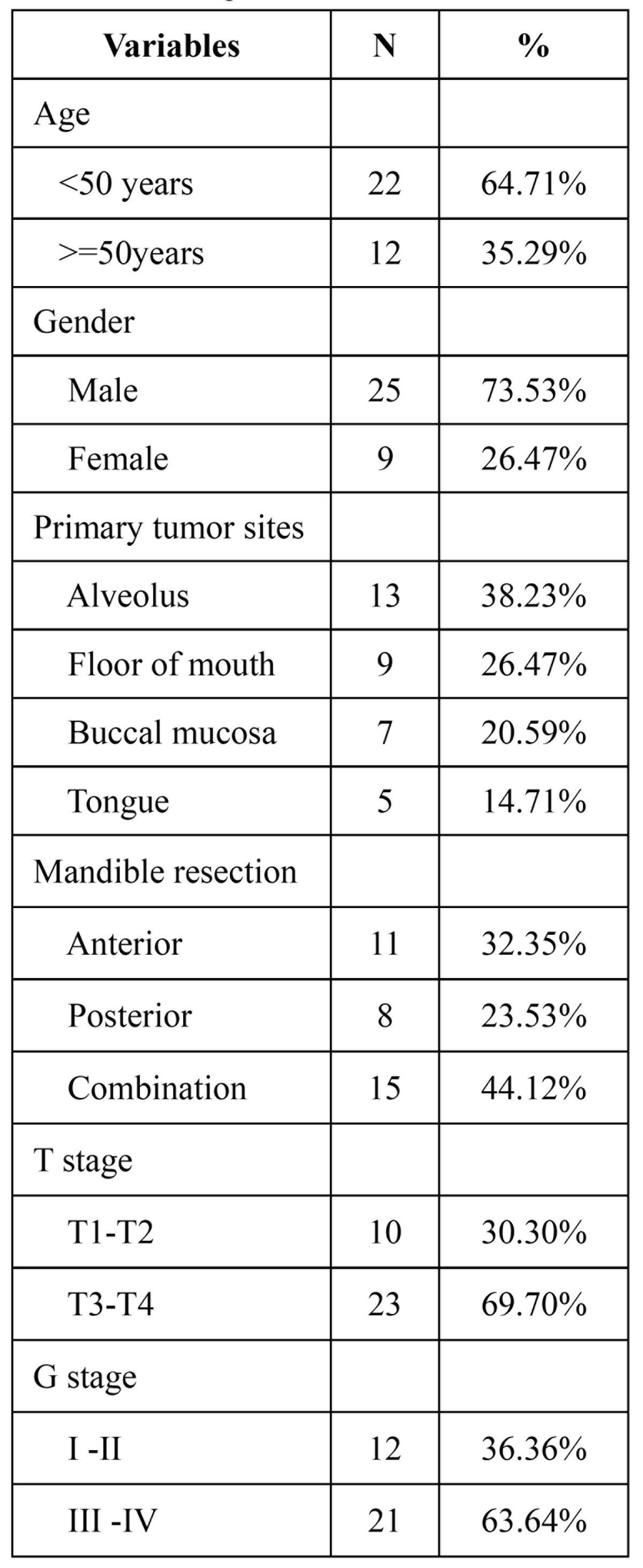
Patients profile.

The postoperative follow-up period ranged from 12 months to 4 years, and the mean follow-up point was 2.3 years. 24 patients were between 1 and 3 years after treatment and the remaining 10 patients had been treated more than 3 years before.

- Quality of life

UW-QOL: The scores for 12 disease-specific domains and the importance of each domain are shown in  Table 2. The best-scoring domain was mood, with an main score of 73.36. The worst score of the domains are chewing and saliva, with the main score of 33.13 and 44.83. The selection of the most important of the three domains, chewing was considered most important projects over the past 7 days followed by speech and swallowing after allowing for patients to choose up to three domains recreation, shoulder and mood domains were considered least important to patients.

**Table 2:**
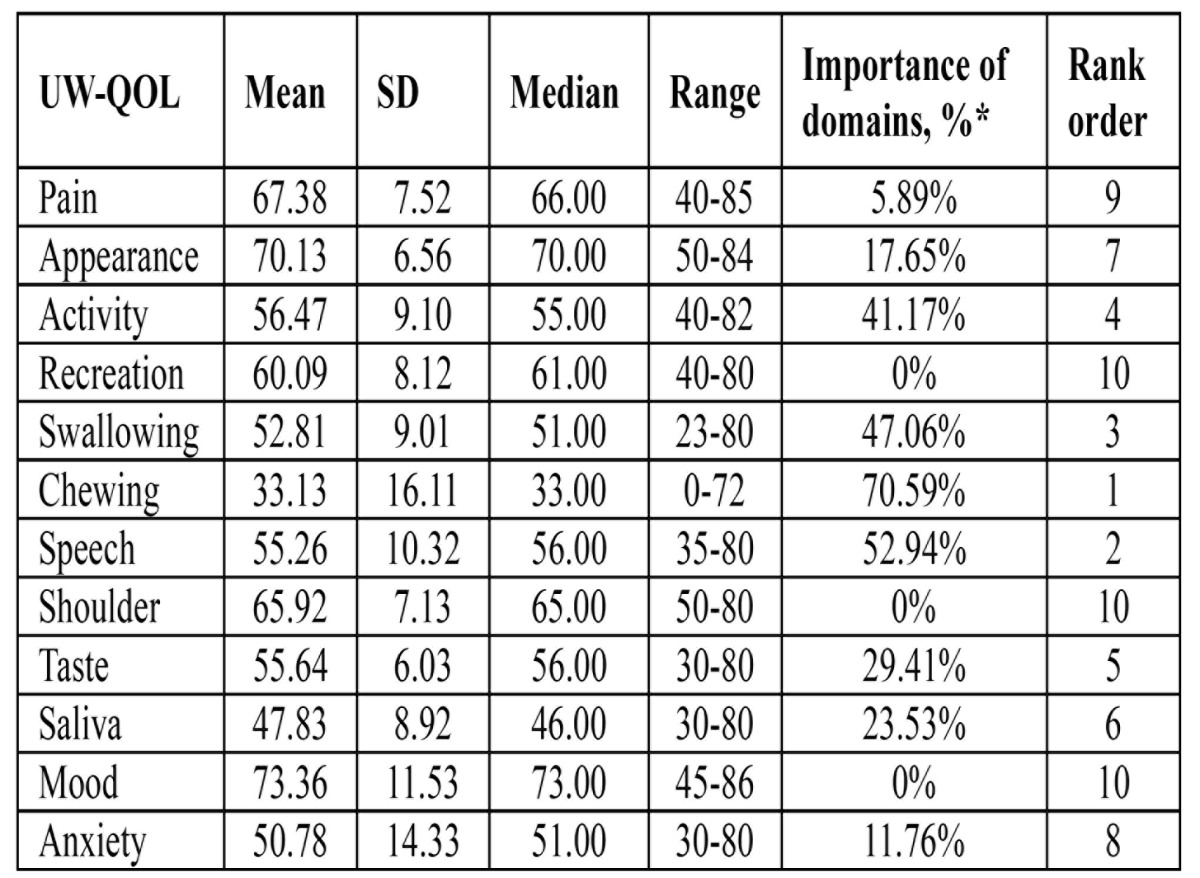
Means of scores of items and scales of UW-QOL questionnaire.

OHIP-14: Distributions of OHIP-14 domain scores at presentation are shown in  Table 3. The best domain scores for the complete group were 35.52 for social disability, 36.33 for handicap, and 45.27 for psychological disability. The highest score was for physical disability and physical pain.

**Table 3:**
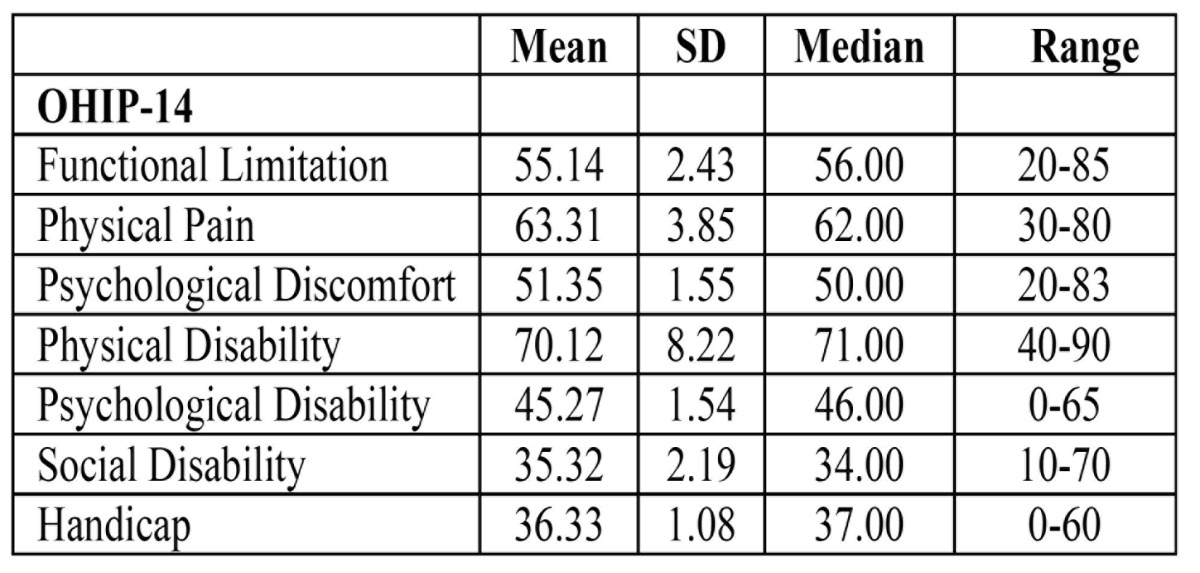
Means of scores of items and scales of OHIP-14 questionnaire.

About sixty percent patients had had little education. Three (8.82%) patients did not complete primary education. Sixteen patients (47.06%) had completed an elementary school and junior middle school education, only two patients (5.88%) had reached university graduation. Thirteen patients (38.24%) had graduated from a senior middle school. Three patients could not read or write, and needed help to complete the questionnaire.

## Discussion

HRQOL should be considered as part of the overall process of care for oral cancer patients. Oral cancer has a profound impact on the quality of life for patients and their families (8). Mandibular bone defects can cause asymmetry, facial disharmony, and tooth loss compromises chewing. The mandible plays a major role in airway protection and support of the tongue, lower dentition, and the muscles of the floor of the mouth permitting mastication, articulation, deglutition, and respiration (9). Reconstruction of mandibular defects after tumor resection is one of the most challenging problems facing reconstructive surgeons. The free fibula flap as a source of vascularized bone in reconstructive surgery is in wide use (10). The fibula has been demonstrated to be an ideal flap for mandibular reconstruction. This is particularly true when a limited number of fibular osteotomies is needed to provide appropriate bone shape (11).

The expectation of clinical outcome of reconstruction is regarded to be the most important factor in the decision, and HRQOL measurement provides information about perceptions of patients (12). HRQOL has recently become a constant preoccupation in the assessment of any therapy in oncology. The great number of questionnaires specific for diseases of the oral cavity reflects that there is no “gold standard”. Our research is using the 14-item Oral Health Impact Profile (OHIP-14) and the University of Washington Head and Neck Quality of Life questionnaire (UW-QOL). We carried out this study to determine the postoperative HRQOL of these patients and the possible relationship of reconstruction surgery.

The oral speciﬁc questionnaire was able to better demonstrate the changes in quality of life due to surgery. Many scholars have chosen to use the UW-QOL questionnaire (6,7). The UW-QOL measure was chosen as the head and neck specific questionnaire because it is short and easy for patients to complete themselves, thus making it ideal in a busy outpatient setting. We can see that the highest score of UW-QOL subscales in present study was in mood domain. The average score was 73.36±11.53, which indicated a slight damage in the mood domain. Besides mood domains in UW-QOL questionnaire, patients scored high in pain (67.38±7.52) and appearance (65.92±7.13) domains, this indicates that mandible reconstruction with free fibula flap have little effect on pain domain. A remarkable finding was that the relatively low scores of UW-QOL subscales in this study were in speech and swallowing domains. The average scores were 55.26±10.32 and 52.81±9.01, which indicated that mandible immediately reconstruction with free fibula flap have bad effect on speech and swallowing function. At the same time we found that patients satisfied with the appearance domains. This may be due to free fibula flaps has provided our with the opportunity to more carefully address the aesthetic and functional reconstruction of mandible defects based on the wide variety of bone and soft tissue available. Thereby obtaining a better facial appearance. However, a significant result was that the lowest score of UW-QOL was in chewing (33.13±16.11) domain. This is may be due to mandible defects caused some teeth lost, thus less chewing function.

Rogers (13) and Chin (14), in their study on importance-rating using the UW-QOL questionnaire in patients treated by primary surgery for oral cancer found that patients tended to rate speech, chewing, and swallowing as more important than the other UW-QOL domains. However, in present study found the different results: chewing, speech and swallowing. This is may be due to in our study, immediate dental implants was positioned in only one patients. So, patients will lose some teeth.

The Chinese version of the OHIP-14, which has been translated and validated for use in Hong Kong and China, was used in this study (7,15). The OHIP was designed to provide a comprehensive measure of the dysfunction, discomfort, and disability attributed to oral conditions. The OHIP-14 consists of 14 items organized into 7 subscales that assess how oral health can affect physical and social wellbeing. In addition the patient can complete it in 10 min. In present study, the best domain scores for the complete group were 35.32 for social disability, 36.33 for handicap, and 45.27 for psychological disability. The highest score was for physical disability (70.12±8.22). This shows that oral cancer surgery does seem to have an overall effect on oral health. Patients believe that surgery has brought a lot of damage to their oral function.

In present study, questionnaires do not contain a section on the effect of the free fibula flap donor site on HRQOL and function. The immediate postoperative donor site morbidity is generally considered to be low and is reported to be in a range between 15% and 55% (16,17). In our series, only three patients exhibited complicated wound healing at the donor site. And 11.76% of the patients reported ankle instability that impaired physical activity, such as walking, running or climbing stairs.

Some studies shown that after adjuvant radiotherapy, compared with operation alone, weight, salivary function, and physical function were significantly reduced and that swallowing, coughing, and symptoms of dry mouth increased (18,19). 20/33 patients in our study (60.61%) who were given postoperative radiotherapy or chemotherapy,8 patients said their oral function decreased after chemotherapy or radiotherapy treatment.

There were several limitations of this study. First, the sample size was small and may not have had sufficient power to find more valuable results. Second, we described oral cancer in the study population at one point in time, and so could not fully assess its impact on patients’ HRQOL over the whole postoperative period.

## Conclusion

Oral cancer patients after mandible immediately reconstruction with free fibula flap would have significantly influenced on the patients’ quality of life, especially in patient’s oral functions.In future oral cancer treatment, HRQOL should be acknowledged as an important outcome parameter, along with the traditional biomedical outcomes. Clinically, HRQOL should be used as part of oral cancer treatment. The sociocultural data showed a rather low education level and standard of living for the most of the patients.
